# Insights on a putative aminoacyl-tRNA-protein transferase of *Leishmania major*

**DOI:** 10.1371/journal.pone.0203369

**Published:** 2018-09-12

**Authors:** Rohit Sharma, Monica Cristina Terrão, Felipe Freitas Castro, Reinhard Breitling, Vitor Faça, Eduardo Brandt Oliveira, Angela Kaysel Cruz

**Affiliations:** 1 Department of Cell and Molecular Biology, Ribeirão Preto Medical School, University of São Paulo, Ribeirão Preto, SP, Brazil; 2 Jena Bioscience GmbH, Jena, Germany; 3 Department of Biochemistry and Immunology, Ribeirão Preto Medical School, University of São Paulo, Ribeirão Preto, SP, Brazil; Instituto Oswaldo Cruz, BRAZIL

## Abstract

The N-end rule pathway leads to regulated proteolysis as an adaptive response to external stress and is ubiquitous from bacteria to mammals. In this study, we investigated a gene coding for a putative core enzyme of this post-translational regulatory pathway in *Leishmania major*, which may be crucial during cytodifferentiation and the environment adaptive responses of the parasite. Leucyl, phenylalanyl-tRNA protein transferase and arginyl-tRNA protein transferase are key components of this pathway in *E*. *coli* and eukaryotes, respectively. They catalyze the specific conjugation of leucine, phenylalanine or arginine to proteins containing exposed N-terminal amino acid residues, which are recognized by the machinery for the targeted proteolysis. Here, we characterized a conserved hypothetical protein coded by the LmjF.21.0725 gene in *L*. *major*. *In silico* analysis suggests that the LmjF.21.0725 protein is highly conserved among species of *Leishmania* and might belong to the Acyl CoA-N-acyltransferases (NAT) superfamily of proteins. Immunofluorescence cell imaging indicates that the cytosolic localization of the studied protein and the endogenous levels of the protein in promastigotes are barely detectable by western blotting assay. The knockout of the two alleles of *LmjF*.*21*.*0725* by homologous recombination was only possible in the heterozygous transfectant expressing LmjF.21.0725 as a transgene from a plasmid. Moreover, the kinetics of loss of the plasmid in the absence of drug pressure suggests that maintenance of the gene is essential for promastigote survival. Here, evidence is provided that this putative aminoacyl tRNA-protein transferase is essential for parasite survival. The enzyme activity and corresponding post-translational regulatory pathway are yet to be investigated.

## Introduction

Leishmaniasis is a vector-borne neglected tropical disease (NTD) that affects large populations worldwide and is caused by various species of the protozoan pathogen *Leishmania*. The disease is highly prevalent, with 12 million cases distributed in more than 80 countries and an estimated population of 350 million at risk of infection. Two million new cases are reported annually [1]. Survival of *Leishmania* in mammalian hosts depends on the differentiation of the invading motile metacyclic promastigotes to the intracellular amastigote forms. This process is the result of the differential expression of several developmentally regulated genes, in an adaptive response to the drastic environmental changes within the phagolysosome of macrophages [2–5]. As a member of the Trypanosomatidae family, *Leishmania* diverged early in eukaryotic evolution, and the regulation of gene expression in these parasites occurs predominantly at the post-transcriptional level. Transcription is constitutive, and genes are organized in long directional clusters transcribed as polycistrons, which are processed by coupled trans-splicing and polyadenylation [6–9].

Although the post-transcriptional regulatory mechanisms utilized by *Leishmania* during the complex developmental transitions have been thoroughly investigated by various research groups, there is much to be explored in terms of the particular roles of post-translational events, such as protein modification and stability [10–11]. We have recently shown that arginine methylation of targeted proteins driven by a protein arginine methyltransferase takes part in the control of parasite virulence [12], and several research groups are currently investigating various modes of post-translational modification [13–16].

The N-end rule pathway described in many eukaryotes and prokaryotes sits among the “last minute” regulatory mechanisms that occur at a post-translational level in response to stress stimuli [17–19]. This pathway regulates the proteolysis of targeted proteins by altering the N-terminal amino acid residue; this modification acts as the primary degradation signal and is referred to as the N-degron. In bacteria, the route is triggered after the conjugation of leucine or phenylalanine amino acids to the target protein carrying a lysine or an arginine as an N-terminal residue. The transfer of the amino acid to the N-terminus is catalyzed by a leucyl-phenylalanyl-tRNA protein transferase (L/F transferase). Interestingly, recent investigations of plants have provided important roles for this pathway in stress-induced conditions such as hypoxia and starvation [20, 21]. In *Plasmodium falciparum*, a sequelog of L/F transferase has demonstrated activity in the eukaryotic ATE1-encoded arginyl-transferases, which conjugate Arg to the N-terminal Asp, Glu or oxidized Cys residue of a target protein. The modified protein is subsequently recognized by ubiquitin ligase and targeted to the proteasome for degradation [22].

Currently, there is no available information to ascertain the presence of such a pathway in *Leishmania*, but in this work, we describe the identification and partial characterization of a gene encoding a hypothetical protein LmjF.21.0725 that carries the signature domain belonging to the Acyl CoA-N-acyltransferases (NAT) superfamily. Among the members of NAT superfamily, the leucyl, phenylalanyl tRNA-protein transferase-like protein (LFTR-like) family is a close relative of the nonribosomal peptidyltransferase [23]. This is the first study suggesting the presence and essentiality of a NAT superfamily homologue in the *Leishmania* parasite. The work raises the possibility of the existence of the N-end rule pathway in *Leishmania major* and may provide a basis for the future characterization of this pathway role in the parasite adaptation and survival within the mammalian host.

## Materials and methods

### *In silico* analysis of LmjF.21.0725 in *L*. *major*

The sequence of the hypothetical conserved protein LmjF.21.0725 from the *L*. *major* strain Friedlin was retrieved from TriTrypDB (http://tritrypdb.org) [24]. InterProScan 4 (http://www.ebi.ac.uk/Tools/pfa/iprscan/) [25], an online protein functional analysis tool, and PANTHER (Protein ANalysis THrough Evolutionary Relationships; http://www.pantherdb.org/) were used to search for a protein signature motif using the respective plugins within Geneious software (Biomatters Ltd, New Zealand) [26, 27]. Alignment of the derived amino acid sequences from the different parasites was performed using the ClustalW program in the BioEdit software package (Version 7.0.4.1) and Geneious software [28]. The phylogenetic tree was constructed using various algorithms (maximum likelihood tree, UPGMA tree and neighbor joining) from the MEGA 7 package [29].

The three-dimensional structure of LmjF.21.0725 was predicted by the Phyre2 server using the default parameters. The Phyre2 server predicts the 3D model from the single submitted protein sequence by gathering homolog sequences from a large non-redundant database, followed by a secondary structure prediction (Hidden Markov Model (HMM)). Multiple alignment and secondary structure are combined and used to predict the structure of the protein and the top scoring alignments are used to construct the “crude backbone-only model”. Loop modeling is used to correct insertions and deletions and amino acid side chains are added at the final Phyre2 predicted structure [30]. The predicted 3D structures were visualized by the UCSF Chimera tool program [31]. PyMOL was used to superimpose the predicted model and the template using the “super” command to calculate the C_α_ backbone root-mean-square deviation (RMSD) [32]. TM-align server was used to determine the TMscore (0.0 < TM-score < 0.30—random structural similarity; 0.5 < TM-score < 1.00, in about the same fold). The server determines the homology between predicted structure and template by applying an algorithm less sensible for the small local deviation in the structure and more sensitive to the global topology [33]. Hydrophobicity surface for the predicted model was determined by “color_h” script of PyMOL software [32].

### Prediction of immunogenic peptide and synthesis

The predicted amino acid sequence of the *LmjF*.*21*.*0725* gene product was analyzed to select for potential antigenic peptides. The initial prediction of hydrophobicity and alpha helicity was performed with software available online (http://web.expasy.org/protscale/) [34]; one of the putative antigenic peptides indicated in S3 Fig, LKKEAAIQRKAEIR, was then manually assembled on Rink amide resin at the 100 μmol scale using the HBTU/HOBt activation protocol for Fmoc solid-phase peptide synthesis [35]. In addition, an N-terminal Cys residue was added to the peptide during the synthesis to facilitate its subsequent coupling to a carrier protein or immobilization onto an activated solid support.

### Raising anti-LmjF.21.0725 antibody

Antibodies aimed at the LmjF.21.0725 protein were raised against its peptide fragment LKKEAAIQRKAEIR, which contained an additional N-terminal Cys residue. Prior to the immunization of rabbits, the peptide was conjugated to Sepharose-BSA. Briefly, 20 mg of bovine serum albumin (BSA) was attached to 2 ml of 1,1’-carbonyldiimidazole-activated Sepharose CL-6B resin, as previously described [36]; the immobilized protein was then activated with 50 mg of bromoacetic acid N-hydroxysuccinimide ester, whose alkylating bromoacetic moiety became available for the coupling reaction with 10 mg of the thiol-containing CLKKEAAIQRKAEIR peptide. Two male rabbits of approximately 1 kg were immunized with the peptide following the laboratory's optimized protocol. Briefly, 250 μg of the conjugated peptide was allowed to swell in 1 ml of phosphate-buffered saline, pH 7.2, and emulsified with 1 ml of complete Freund’s adjuvant; each animal received approximately 1 ml of emulsion, injected subcutaneously in 6–8 different spots. Two booster doses of the conjugated peptide emulsified with normal Freund’s adjuvant were given 30 and 45 days after primary injection, respectively. One week after the secondary booster, 5 ml of venous blood was drawn from the rabbit's ear, and the corresponding serum was titrated against the peptide by ELISA, as described [37], pre-immune sera from rabbit was included as a negative control.

### Ethical statement

The use of rabbits was approved by the Commission of Ethics in Animal Research (CETEA) at the Ribeirão Preto Medical School, University of São Paulo. They certified that Protocol n° 118/2008 (“Control of gene expression and genetic plasticity in *Leishmania*”) is consistent with the ETHICAL PRINCIPLES IN ANIMALRESEARCH adopted by Brazilian College of Animal Experimentation (COBEA) in 8/27/2012.

### Affinity purification of anti-LmjF.21.0725 antibody and western blotting

The purification of the anti-LmjF.21.0725 antibody was achieved by a two-step procedure: a crude IgG fraction was prepared by slow addition, under stirring, of 5 ml of saturated ammonium sulfate solution to 10 ml of the rabbit immune serum, followed by centrifugation at 500 x g for 10 min. The precipitate was recovered, dissolved in 0.02 M Tris-buffered saline, pH 8.1, and dialyzed to remove excess salt. Further purification was achieved by percolating the crude IgG solution through a peptide-Sepharose column under affinity chromatography conditions. The affinity column was synthesized by overnight incubation, at room temperature, of 10 mg of the CLKKEAAIQRKAEIR peptide in 5 ml of 0.5 M sodium bicarbonate with 5 ml of butenediol diglycidyl ether-activated Sepharose 4B. After extensive washing of the column with 50 ml of Tris-buffered saline, antibodies were eluted with 0.2 M glycine buffer, pH 2.8. The affinity purified anti-LmjF.21.0725 antibody was stored at -20°C in Tris-buffered saline containing 30% glycerol and 1% BSA.

The western blotting conditions using anti-LmjF.21.0725 antibodies were optimized by separating the cell lysate proteins from *L*. *major* by electrophoresis on SDS-PAGE gels and then subjecting strips of the corresponding nitrocellulose membrane to various dilutions of the affinity-purified anti-LmjF.21.0725 antibody. As a result, in the western blotting experiments performed in this work, nitrocellulose membranes with transferred proteins were treated with blocking solution (10% defatted milk and 0.1% tween-20 in phosphate-buffered saline) overnight and probed with anti-LmjF.21.0725 (1:6000 in blocking buffer, 8 hours), followed by incubation with sheep anti-rabbit-IgG conjugated to horseradish peroxidase (1:25000 in blocking solution, overnight) as the secondary antibody. For signal detection, we used ECL western blotting detection reagents (GE Healthcare, Buckinghamshire, UK).

### Construction of vectors for knockout and overexpression of *LmjF*.*21*.*0725* gene and transfection conditions

Knockout of both of the alleles encoding LmjF.21.0725 (indicated as *LmjF*.*21*.*0725*) were attempted via homologous recombination. Two vectors bearing antibiotic resistance markers flanked by the 5’ and 3’ regions of the targeted CDS were constructed to replace both alleles from the wild-type *L*. *major* CC1 strain (Part A of S1 Fig). The *LmjF*.*21*.*0725* locus was amplified from the genomic DNA, including the CDS (1131 bp) and ~600 nucleotides each from the up- and downstream sequences, based on the sequence deposited at TritrypDB; primers were designed (http://bioinfo.ut.ee/primer3-0.4.0) to generate the amplicon (S1 Table). Two unique restriction sites for enzymes *Bcl*I and *Ale*I were used to delete the ORF from the plasmid. The original CDS of *LmjF*.*21*.*0725* in the plasmid was replaced by hygromycin phosphotransferase and puromycin phosphotransferase genes, which are the resistance markers for hygromycin B (HYG) and puromycin (PAC), respectively.

The resistance markers HYG and PAC were amplified from vectors available in the laboratory using the specific primers with *Bcl*I/*Ale*I restriction sites (S1 Table). Amplicons were then cloned into the pGEMT vector (Promega, USA). The recombinants, pGEMT_5FR_*LmjF*.*21*.*0725*_3FR, pGEMT_PAC and pGEMT_HYG, were confirmed by Sanger sequencing. The final constructs used for the KO experiments—pGEMT_5FR-HYG-3FR and pGEMT_5FR-PAC-3FR—were also confirmed by sequencing. The linearized KO cassette for PAC and HYG was obtained by *Not*I and *Apa*I-*Stu*I double digestion, respectively (Parts B and C of S1 Fig). *L*. *major* CC1 was transfected with the linear targeting fragments by electroporation [38]. Briefly, 2–5 μg of the purified linear fragment was used to transfect the parental cell line (+/+), and transfected cells were plated onto M199 containing the appropriate antibiotics (hygromycin B or puromycin).

To construct a plasmid for the exogenous expression of LmjF.21.0725, the CDS and flanking regions of this gene (2.4 kb) were amplified with the designed primer (S1 Table) and cloned between *Bst*EII and *Xma*I restriction sites of the *Leishmania* expression vector pXNeoGFP. Green Fluorescent Protein (GFP) was used to monitor the presence of the plasmid by FACS. The recombinant, pXNeoGFP-*LmjF*.*21*.*0725* (Part A of S4 Fig), was confirmed by restriction map diagnostics and sequencing. The heterozygous line (+/PAC) was transfected with pXNeoGFP-*LmjF*.*21*.*0725* (5 μg) to generate the +/PAC(+) line; transfectants were selected with G418 and puromycin and analyzed for expression of LmjF21.0725 by western blotting.

Individual clones of +/PAC/(+) and conditional null mutant, HYG/PAC/ (+) were isolated from the M199 solid medium (2% agar plates). The media were supplemented with each drug or a combination of puromycin, hygromycin B and G418 in concentrations corresponding to 4 x IC_50_ each. Four independent clones were isolated and expanded in liquid medium. A heterozygote carrying the pXNeoGFP vector, named [+/PAC (V)], was used as a control line.

### FACS

GFP expression level was monitored using a FACSCalibur flow cytometer (BD Biosciences). Parasites (2x10^7^ promastigotes) were washed twice in PBS/FBS (1%). An equal number of *L*. *major* (WT) parasites were used as a negative control, and 100,000 events were recorded for both the *L*. *major* transfectant and wild-type parasites. Promastigotes from all transfectants were harvested (3x10^7^ cells) after ten passages in the absence of drug pressure.

### Subcellular localization in the promastigote stage of *L*. *major*

For the subcellular localization of LmjF.21.0725, promastigotes of *L*. *major WT* and +/PAC/(+) transfectant (2x10^7^ cells, each) were pelleted and washed twice in PBS. Poly-lysine coated slides (0.01%) were prepared, and parasites were diluted to 1:10 and microscopically examined. The parasites were applied to the slides after fixation in 3% formaldehyde; non-adherent cells were removed by a gentle wash with PBS. The slides were blocked with 2% BSA, 0.1% Triton X-100 in PBS for 1 hour, followed by incubation with anti-LmjF.21.0725 (1:500 in blocking solution) for 1 hour. The cells were washed three times with PBS and then incubated with F (ab') 2-Goat anti-Rabbit IgG (H+L) Secondary Antibody conjugated with Alexa Fluor (1: 400 in blocking solution) for ½ hour. The cells were washed three times with PBS, and a sufficient quantity of DAPI was applied. After washing the slides three times, coverslips were applied, and the slides were left for 24 h, at 4°C.

### Southern blot and PFGE analysis of transfectants

To analyze the correct recombination events and to check for the presence/absence of endogenous *LmjF*.*21*.*0725*, we performed southern blotting analysis. Briefly, we used 10 μg of genomic DNA from the following *L*. *major* lines: wild type/parental (+/+); heterozygous (+/PAC); heterozygous with the LmjF.21.0725 expressing vector (+/PAC/(+)) and a conditional transfectant HYG/PAC/(+) digested with *Sal*I and fractionated in 0.8% agarose gel. Blotted nylon membranes were probed with the DNA fragments internal to the coding sequences for hygromycin phosphotransferase (HYG, 690 bps), puromycin acetyl transferase (PAC, 512 bps) or *LmjF*.*21*.*0725* (472 bps). Hybridization and washing steps were performed following the manufacturer’s instructions (AlkPhos Direct Labeling and Detection System, GE Healthcare Life sciences, USA).

Genomic DNA from the studied strains was embedded in low-melting-point agarose blocks treated with proteinase K, as previously described [39]. Electrophoresis was performed in 1% agarose gel for 48 hours with initial and final switch times of 360 and 800 seconds, respectively (4.5 V/cm; 16°C; 1x TBE buffer).

### Expression of C-terminal hexa-histidine tagged LmjF.21.0725 in *Leishmania tarentolae*

The pLEXSY_NEO2 expression vector (Jena Bioscience GmbH, Germany) was employed for intracellular expression of LmjF21.0725. The construct was designed for the targeted integration of the *LmjF*.*21*.*0725* cassette into the ribosomal *SSU* locus of the genome of *L*. *tarentolae*, a non-pathogenic host useful for overexpression of *Leishmania* proteins [40]. The CDS of *LmjF*.*21*.*0725* was cloned between *Bgl*II and *Nhe*I (primer sequence, S1 Table). Approximately 10 μg of the pLESXY_NEO_*LmjF21*.*0725* HIS circular construct was linearized with *Swa*I and ethanol-precipitated. The fragment was suspended in 50 μl of water and 350 μl (ca. 4x 10^7^ cells) of prechilled *L*. *tarentolae* cultured cells were transfected. Electroporation was performed in a pre-chilled 2-mm cuvette (450 V and 450 μF, Genepulser, BIORAD) using a time range of 5.5–5.6 ms/pulse. The electroporated cells were further incubated on ice for 10 minutes and then transferred to 10 ml of BHI medium for 20 hours. The cells were gently dispersed on BHI agar plates supplemented with G418 (50 μg/ml). After ten days of incubation at 26°C in the dark, colonies (1–2 mm diameter) were picked and cultured in 1 ml of LEXSY selective media for 2–3 days. The clones were microscopically examined for growth, motility, and shape. To screen for LmjF.21.0725 expression, 500 μl of the densely grown culture were analyzed by Western blotting using specific anti-LmjF.21.0725 and anti-histidine antibodies. The positive clones were then transferred to 10 ml of BHI selective medium and grown to OD 1.4 (48 hours, 26°C and shaking at 140 rpm).

### Protein purification and mass spectrometry analysis (MS)

Purification of the recombinant protein was performed by immobilized metal-affinity chromatography (IMAC) on 1 ml of HIS-Select® Nickel Affinity Gel (Sigma) following the manufacturer's instructions. Cell lysis was performed with a phosphate buffer solution containing 1% NP40, pH 7.4. Elution was performed with a gradient of imidazole concentration from 20 to 500 mM. The purified rLmjF.21.0725-HIS eluted at approximately 320 mM of imidazole. For the MS analysis, the protein was fractionated on 12% polyacrylamide gel (TGX precast polyacrylamide gel, BIORAD) and stained with MS-compatible colloidal Coomassie staining [41]. The gel was destained with several washes of sterile water, and carefully excised gel bands were pooled together for mass spectrometry analysis.

## Results

### *In silico* characterization of LmjF.21.0725

**Fig 1:**
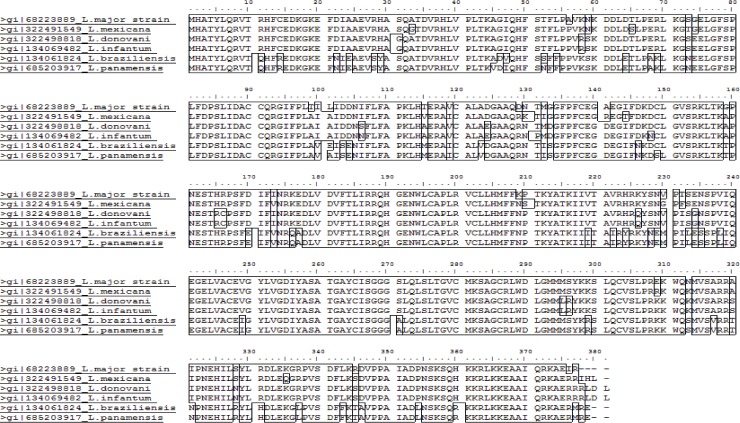
Amino-acid sequence alignment of LmjF.21.0725 homologs derived from various *Leishmania* species.

*LmjF*.*21*.*0725* is annotated as a gene coding for a conserved hypothetical protein of unknown function. A sequence similarity search using BLASTp against the non-redundant protein database (nr) of NCBI resulted in 83%-100% sequence identity with conserved hypothetical protein sequences of various species of *Leishmania* from both subgenera (E-value 0) (Fig 1). Interestingly, this analysis also revealed that LmjF.21.0725 is similar to the assigned putative leucyl, phenylalanyl-tRNA protein transferase sequences of more distantly related species, including *Bodo saltans*, a free-living nonparasitic kinetoplastid flagellate (identity of 45%, E value-2e-79), and *Phytophthora nicotianae* (identity of 25%; E value 2e-10) (S2 Table).

**Fig 2:**
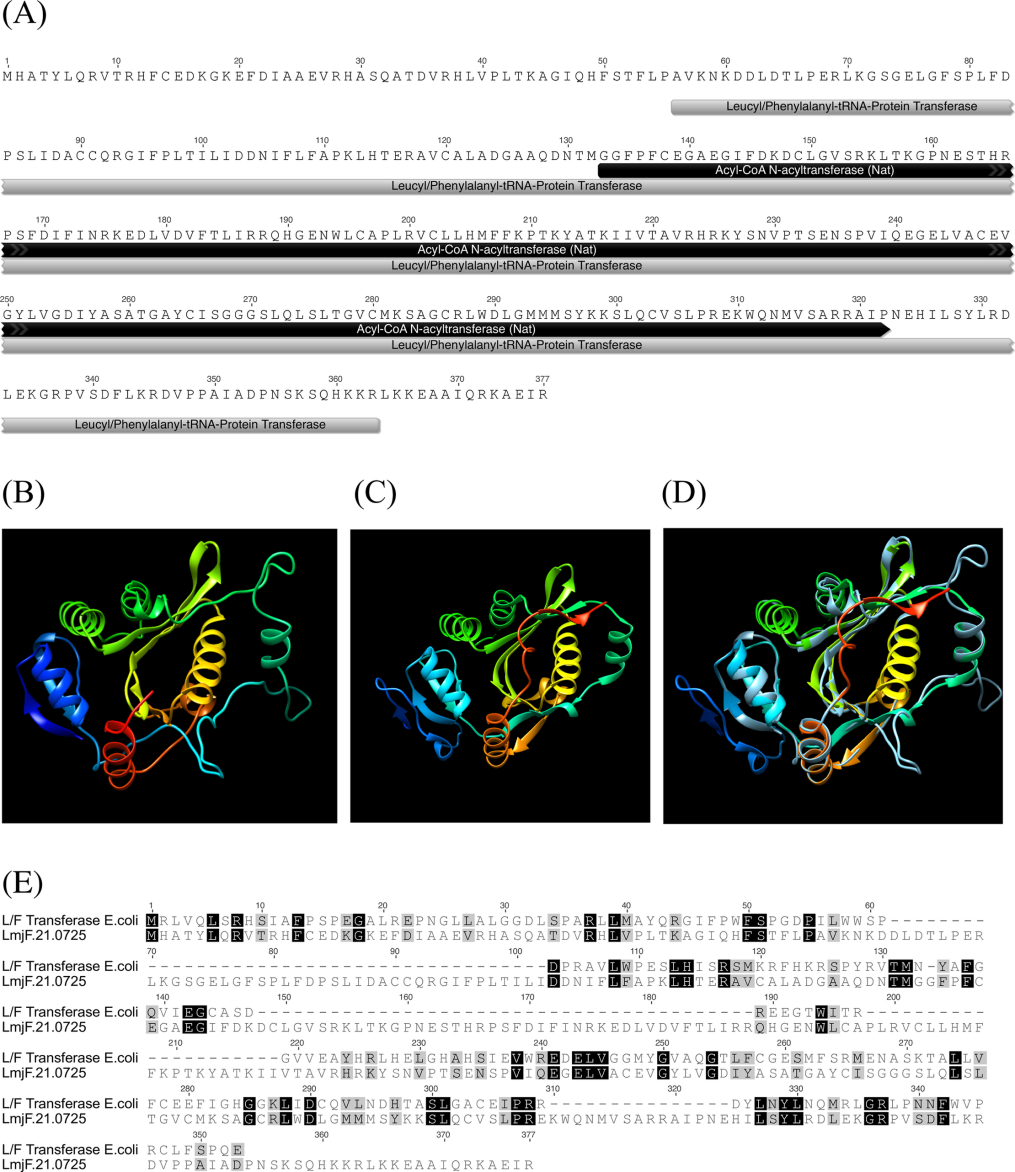
**(A)** PANTHER classification system categorizing LmjF.21.0725 as L/F transferase; InterProScan results showing the signature motif acyl-CoA-N-acyltransferase (NAT) and the predicted amino acid sequence of LmjF.21.0725. **(B)** Three-dimensional structure of LmjF21.0725 predicted by the Phyre2 server. **(C)** Template c2cxA (L/F transferase from *E*. *coli*) **(D)** Superimposed modeled structure of LmjF21.0725 with template c2cxA **(E)** Amino-acid sequence alignment between LmjF21.0725 and L/F transferase from *E*. *coli*.

An analysis conducted to identify the protein signature using the InterProScan tool showed that the protein carries an Acyl-CoA-N-acyltransferase (NAT) superfamily domain between the 133^rd^ and 322^nd^ amino acid positions. In addition, using the PANTHER classification tool, the *Lmj*.*21*.*0725* predicted product was also identified as a putative L/F transferase (Fig 2A). We predicted the three-dimensional structure of Lmj.21.0725 using an online version of the Phyre2 software (Fig 2B). The software predicted the structure based on the available crystal structure of the *Escherichia coli* protein (c2cxA; leucyl, phenylalanyl-tRNA protein transferase, PDB) (Fig 2C). Phyre2 used 52% of the Lmj.21.0725 sequence (192 AA) to generate a model with 100% confidence using the single highest scoring template. Superimposition of the predicted model on the template c2cxa showed significant structural homology (Fig 2D). The determined parameters for the structural homology such as C_α_ backbone root-mean-square deviation (RMSD) and TMscore were 2.575 Å and 0.82431, respectively. Considering the higher possibility of the active sites location on the large pocket of the protein structure, we determined the largest pocket on the predicted structure. Use of the suite “Phyre2 investigator” further confirmed the high quality of the predicted Lmj.21.0725 structure (S2 Fig). Overall, these *in silico* results suggest that LmjF.21.0725 might be a structural homologue of *E*. *coli* L/F transferase, even though it possesses low overall sequence homology (similarity 30%; identity-17%) (Fig 2E).

**Fig 3:**
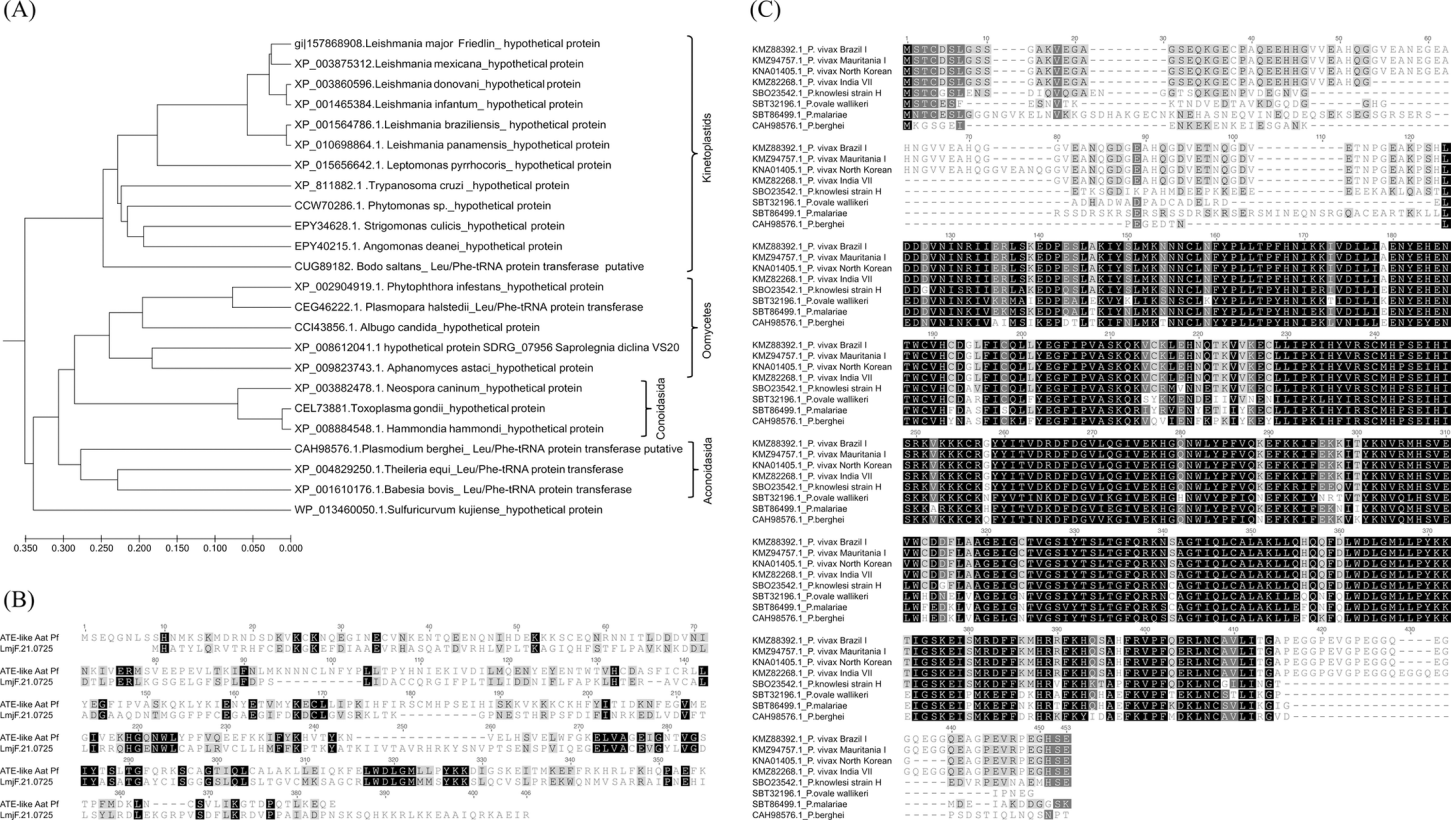
**(A)** Phylogenetic tree based on sequences of 23 selected homologues of LmjF21.0725, constructed by the UPGMA algorithm. **(B)** Pairwise sequence alignment between arg-tRNA protein transferase, e.g., Aat of *P*. *falciparum* and Lmj.21.0725. **(C)** Multiple sequence alignment of putative L/F transferase from various species of *Plasmodium*.

A phylogenetic tree was generated based on the Lmj.21.0725 amino acid sequence and available homologue sequences in the NCBI database. The result of the analysis confirmed the closeness of Lmj.21.0725 with the putative L/F transferase of *B*. *saltans*, a free-living flagellate protozoan, and classified kinetoplastids, oomycetes, aconoidasida and conoidasida as the four separated taxons. Interestingly, the inferred tree also discriminated the *Leishmania* species into the subgenera *Leishmania* and *Viannia* as two distinct monophyletic lines, indicating the Lmj.21.0725 sequence as a potential phylogenetic marker for *Leishmania* spp. (Fig 3A). Interestingly, *P*. *falciparum* has the only functionally characterized aminoacyl tRNA protein transferase among the protozoan parasites; it is a sequelog of an L/F transferase (ABG02898.1) with a catalytic activity of an arginyl-tRNA protein transferase, which is typical in eukaryotes. LmjF.21.0725 showed limited homology with the *P*. *falciparum* enzyme (20% identity, 36% similarity) (Fig 3B). We conducted a comparative sequence analysis of the putative L/F transferase from *Plasmodium* spp and observed high divergence among species. Interestingly, our analysis also indicated amino acid variations of putative L/F transferase within *P*. *vivax* isolates from Brazil, India, Korea and Mauritius (Fig 3C).

### Double knockout of *LmjF*.*21*.*0725* was only possible after adding back a plasmid ectopically expressing the gene

**Fig 4:**
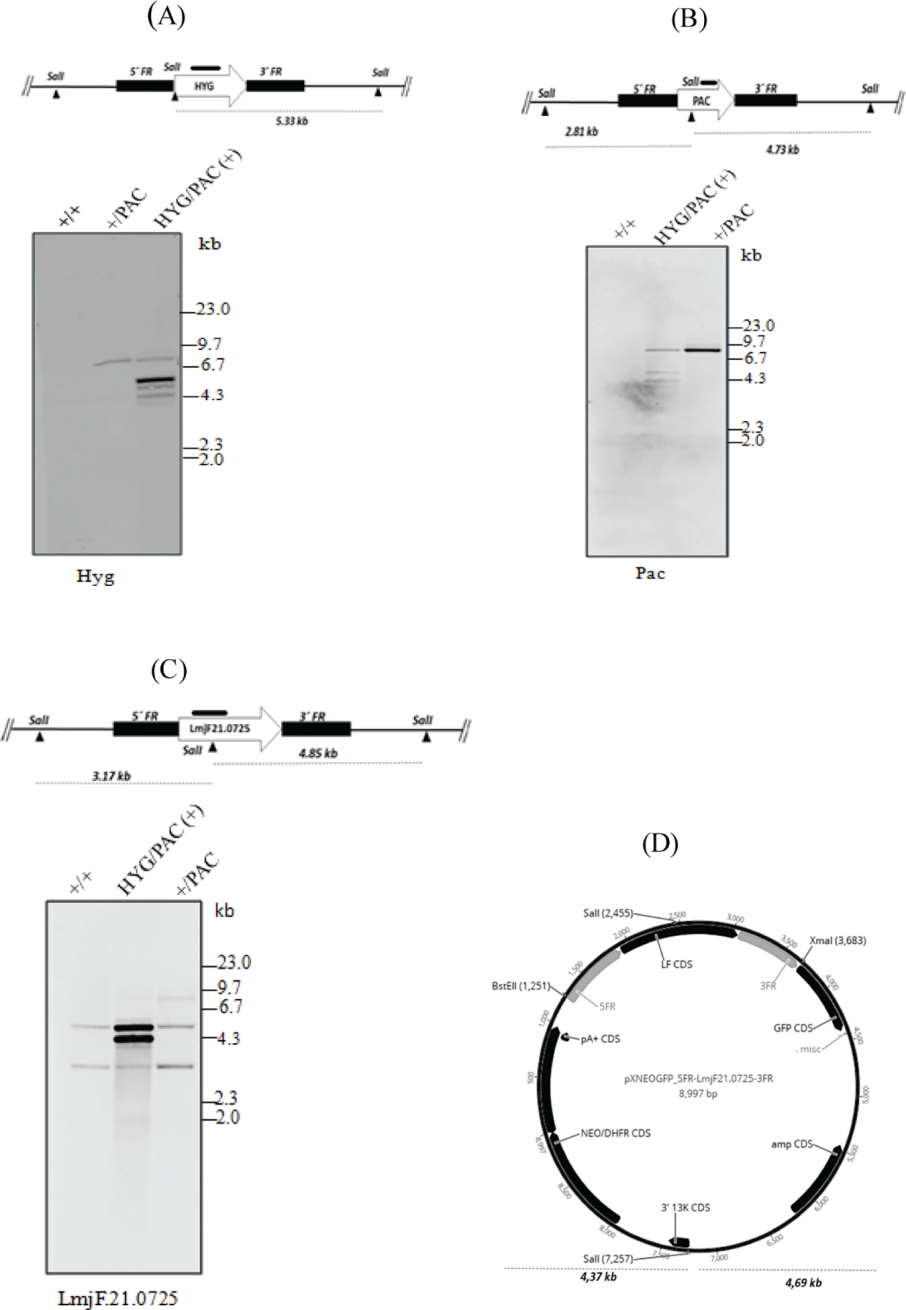
Analysis of the *LmjF*.*21*.*0725* locus anatomy in wild-type and transfectants of *L*. *major*. **(A)** *In silico* physical map of HYG replacement of the endogenous allele is depicted in the cartoon and includes *Sal*I digestion sites (above); expected fragment size of *Sal*I digested DNA is indicated below (interrupted line) and HYG probe fragment is depicted as coarse line above diagram. Southern blotting membrane hybridized with HYG probe is shown below. **(B)** The *in silico* physical map of PAC replacement of endogenous allele is depicted in cartoon and includes *Sal*I digestion sites (above); expected fragment size of *Sal*I digested DNA is indicated below (interrupted line) and PAC probe fragment is depicted as coarse line above diagram. The Southern blotting membrane hybridized with PAC probe is shown below. **(C)** The *in silico* physical map of endogenous *LmjF*.*21*.*0725* allele is depicted in cartoon and includes *Sal*I digestion sites (above); the expected 2 fragments of *Sal*I digested DNA are indicated below (interrupted line), and the *LmjF*.*21*.*0725* probe fragment is depicted as the coarse line above the diagram. The southern blotting membrane hybridized with the *LmjF*.*21*.*0725* probe is shown below. **(D)** Map of pxNeoGFP-LmjF21.0725 used for overexpression of *LmjF*.*21*.*0725*, and *Sal*I digestion predicted fragment sizes are indicated below. *Leishmania* wild-type (WT) and transfectants are indicated in each blot as follows: +/+ = WT; +/PAC = heterozygous line with a PAC cassette replacing one endogenous allele; +/PAC/(+): heterozygous transfectant bearing PAC and plasmid expressing *LmjF21*.*0725* (pxNeoGFP-LmjF21.0725); PAC/HYG/(+) = double replacement transfectant with both selectable markers and plasmid expressing *LmjF21*.*0725* (pxNeoGFP-LmjF.21.0725, addback transfectant). Marker: Lambda DNA-*Hin*dIII digested.

To understand the function of LmjF.21.0725, we planned to delete the two alleles (Fig 4A) from the *L*. *major* genome using linearized KO cassettes (S1 Fig). However, after obtaining a heterozygous line (+/PAC), the double knockout (KO) was not achievable, even after seven independent experiments. Taking the negative result as a possible indication of the essentiality of the gene, we transfected a plasmid expressing LmjF.21.0725 and the GFP as a reporter (pXNeoGFP_*LmjF21*.*0725*, Fig 4D) on the heterozygous line, using the *L*. *major +*/PAC transfectant to generate +/PAC/(+). Another round of transfection to delete the second allele encoding LmjF.21.0725 was then successful, and several independent clones called PAC/HYG/(+) were obtained.

Nevertheless, analysis of the endogenous locus anatomy of the PAC/HYG/(+) transfectant, conducted by southern blotting, suggested that an extra copy of the endogenous gene was present. When using the HYG probe, we confirmed the proper integration of only one allele in the correct locus, and the predicted 5.3-kb band was observed (Fig 4A). Nevertheless, probing with PAC revealed an unexpected architecture in the *LmjF*.*21*.*0725* locus, indicated by a 7.5–8 kb band instead of the predicted 4.7-kb band (Fig 4B). This band could be explained if the original *Sal*I site within the PAC sequence were not functional (Fig 4B), although this site was intact in the used KO cassette as confirmed by sequencing. To confirm deletion of the *LmjF*.*21*.*0725* endogenous copy and distinguish the endogenous gene from the plasmid copy of the gene, an internal fragment from *LmjF*.*21*.*0725* was used as a probe (Fig 4C, upper panel). In this case, we detected the two predicted bands for the endogenous *LmjF*.*21*.*0725* in the WT and heterozygous transfectant (3.2 kb and 4.9 kb), in addition to the *LmjF*.*21*.*0725*-plasmid-derived (pXNeoGFP-LmjF21.0725) bands (4.2 and 4.8 kb, Fig 4C). Nevertheless, in the HYG/PAC (+) cells, we still observed an extra band with a length comparable to the endogenous locus anatomy, with a fainter band co-migrating with the 3.2-kb fragment (Fig 4C).

**Fig 5:**
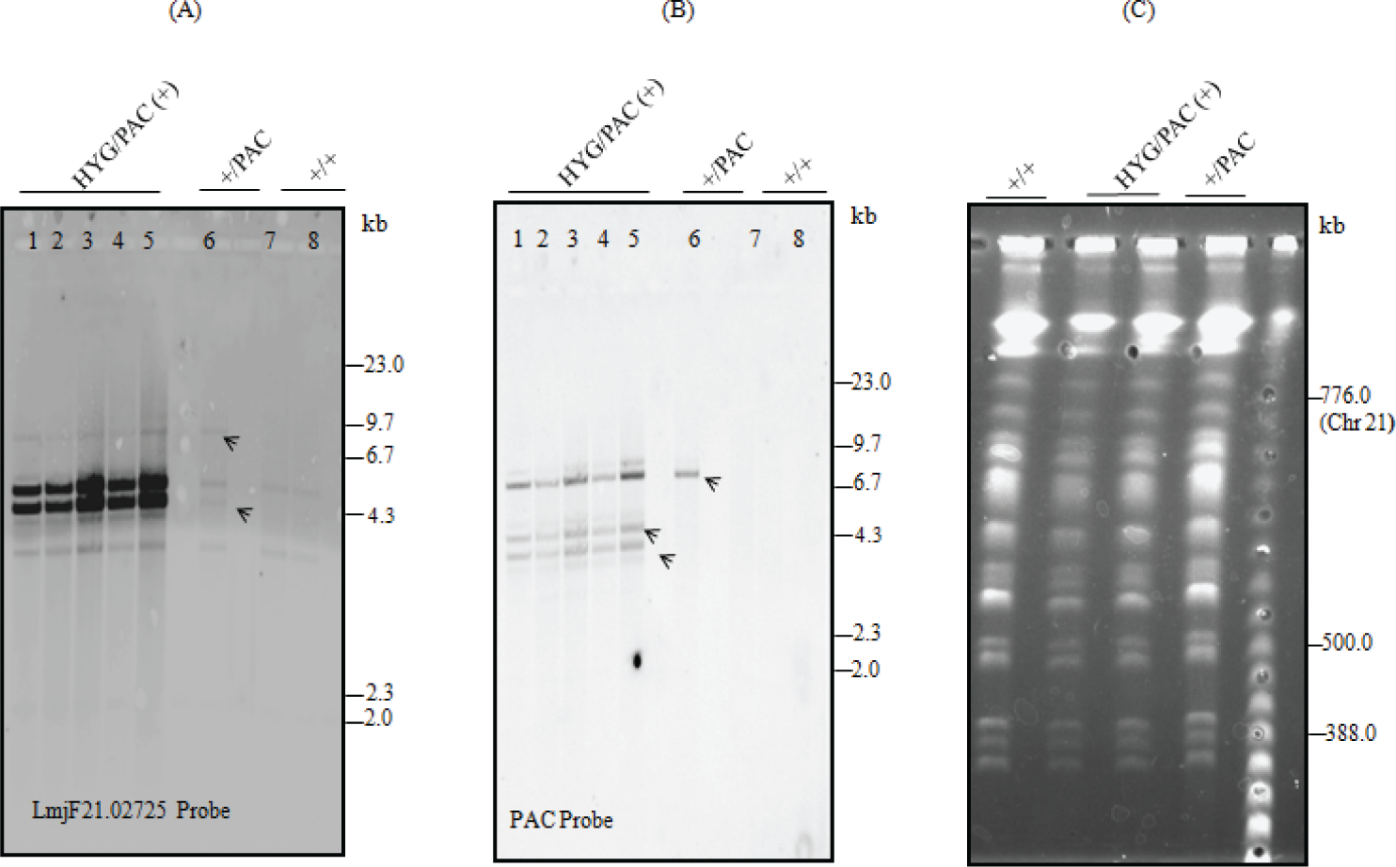
Analysis of the *LmjF*.*21*.*0725* locus structure on independent transfectant clones and comparative analysis of their molecular karyotypes. **(A)** Southern blot of genomic DNA from five different PAC/HYG/ (+) *Leishmania* clones digested with *Sal*I and hybridized with an LmjF21.0725 fragment as probe. Arrows pointing to unexpected bands present in all PAC/HYG/ (+) clones (lane 1 to 5) and in +/PAC (lane 6) cell lines, +/+ (lanes 7 and 8) that revealed the expected locus architecture (lane 7- *L*. *major* CC1; lane 8—*L*. *major* Friedlin). **(B)** Southern blot of genomic DNA digested with *Sal*I and hybridized with PAC-specific probe; arrows indicate unexpected 8 kb bands in all mutants. **(C)** Molecular karyotype of *L*. *major* strains by pulsed field gel electrophoresis. +/+ = WT; +/PAC = heterozygous line with a PAC cassette replacing one endogenous allele; PAC/HYG/(+) = double replacement transfectant bearing both selectable markers and plasmid expressing *LmjF21*.*0725* (pxNeoGFP-LmjF21.0725, addback transfectant). Marker: Lambda ladder (New England Biolabs).

We repeated the southern blot experiment with five randomly selected PAC/HYG/ (+) clones, the heterozygous clone +/PAC and two wild-type *L*. *major* strains. The results with these five independent PAC/HYG/(+) transfectants were also suggestive of the presence of an additional endogenous copy of *LmjF*.*21*.*0725* and an abnormal additional fragment probing for endogenous *LmjF*.*21*.*0725* and PAC marker, respectively (Fig 5A and 5B). The extra bands (indicated by the arrows) present in all transfectants might be a product of either partial digestion or an unexpected recombination event that allowed maintenance of two endogenous alleles and the replacement of one extra allele by PAC. PFGE fractionation of chromosomes from WT and transfectants indicated the lack of marked chromosomal ploidy/somy changes (Fig 5C). However, we cannot exclude possible trisomy or internal chromosome changes, including the gene copy number of the studied locus.

### Experimental proof of *LmjF*.*21*.*0725* essentiality in *L*. *major* promastigotes

**Fig 6:**
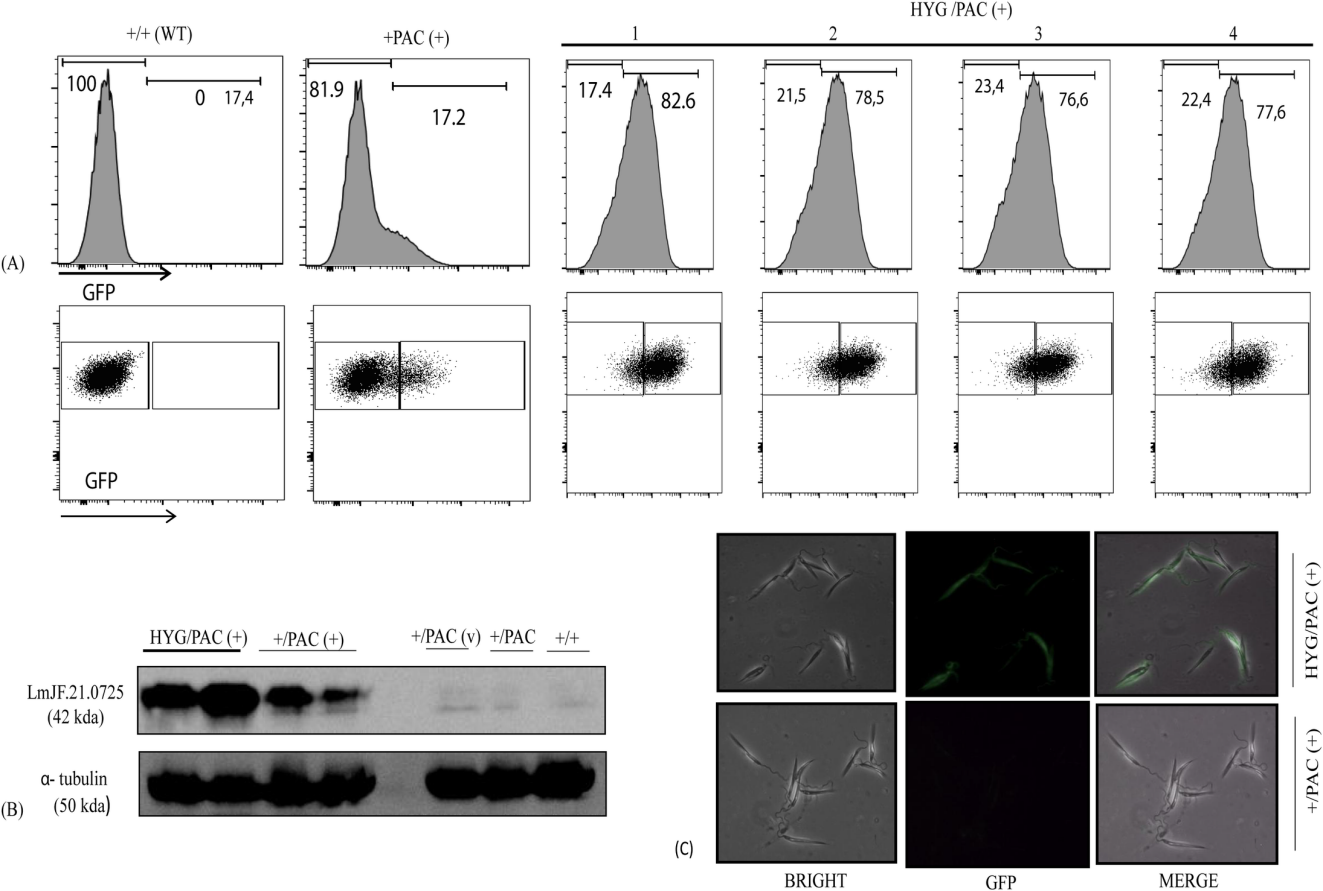
LmjF.21.0725 is essential for *L*. *major* promastigotes. **(A)** FACS analysis was used to quantify GFP levels and estimate dynamics of passive loss of pXNeoGFP-*LmjF*.*21*.*0725* in four independent transfectants in the absence of G418(depicted as numbers 1 to 4, top of the panel). **(B)** With same culture conditions, western blot analysis indicated differences in LmjF.21.0725 expression levels in transfectant lines, using anti-LmjF.21.0725 antibody. **(C)** Fluorescent microscopy images showing GFP expression in transfectants (10^th^ passage in absence of G418). Lines: +/PAC (+) = heterozygous cell line with pXNeoGFP-LmjF21.0725; HYG/PAC(+) = double replacement transfectant bearing both selectable markers and plasmid expressing *LmjF*.*21*.*0725* (pxNeoGFP-LmjF21.0725, addback transfectant).

To aggregate one factor indicative of the essentiality of *LmjF*.*21*.*0725*, the above-described PAC/HYG/ (+) lines were used to evaluate the plasmid maintenance under no drug pressure. We conducted a comparative analysis of the kinetics of plasmid loss on the control cells and PAC/HYG/ (+) transfectants as a measure of the relevance of the plasmid/exogenous gene for the survival of the parasite. The parasites were kept in culture in the absence of drug for ten serial passages and submitted to FACS analysis to examine the dynamics of plasmid loss by monitoring GFP expression levels. We observed that, differently from the control lines, all four tested PAC/HYG/ (+) independent transfectant clones maintained the plasmid bearing exogenous LmjF.21.0725 and GFP (>76% of cells in each culture retained the plasmid). In contrast, the dynamics of plasmid loss of the heterozygous mutants (+/PAC(+)) were fast, and only ~17% of the cells maintained the plasmid after 10 serial passages, as indicated by the distribution of cells with an intense fluorescent signal (Fig 6A). Additionally, the FACs results were confirmed by western blot analysis for Lmj.21.0725 levels, performed on the total protein extracted from independent clones of PAC/HYG/ (+) and +/PAC (+) (Fig 6B), and loss of GFP expression was evidenced by direct fluorescence microscopy images (Fig 6C). In conclusion, the failure to knockout both alleles of *Lmj*.*21*.*0725* and the maintenance of the plasmid in the absence of drug pressure, happening only on the heterozygous line, support the hypothesis that *Lmj*.*21*.*0725* is an essential gene for the survival of *L*. *major* promastigotes.

### Subcellular localization of LmjF.21.0725 in *L*. *major* promastigotes

**Fig 7:**
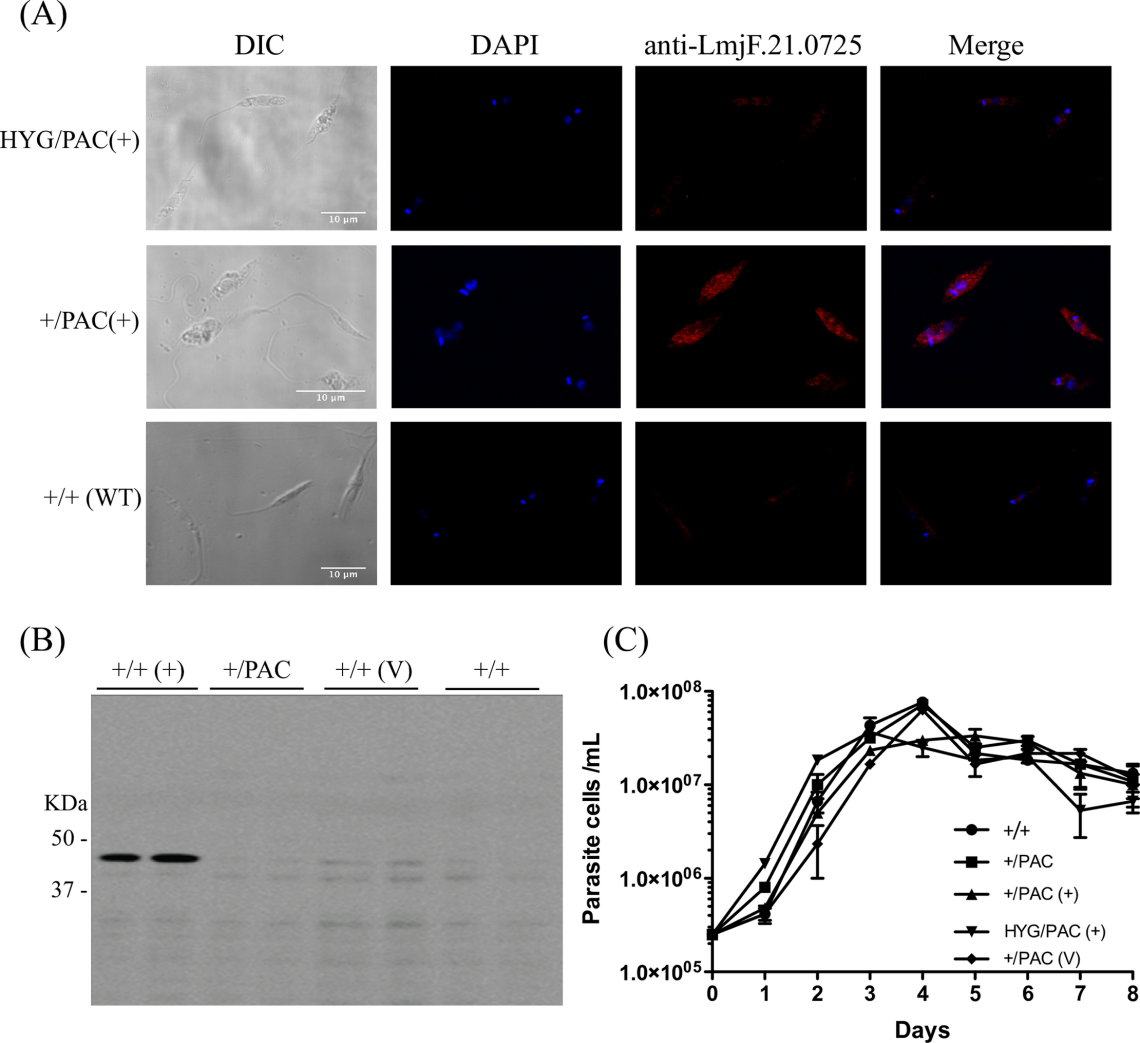
Immunolocalization of LmjF.21.0725 in *L*. *major*. **(A)** Subcellular localization of Lmj21.0725 in *L*. *major* strains: (+/+: WT; +/PAC/ (+): heterozygous cell line with addback pxNeoGFP-LmjF21.0725; PAC/HYG/ (+): Null mutant with addback pxNeoGFP-*LmjF21*.*0725*; Representative cells were visualized with bright field (B/F), DAPI and anti-LmjF.21.0725 antibody overlays. Nuclei and kinetoplasts were stained with DAPI (blue). **(B)** Western blot analysis with anti-LmjF.21.0725 antibody confirming expression of ~42 kDa LmjF.21.0725 in *L*. *major promastigotes (*+/+: WT; +/PAC/ (+): heterozygous cell line with addback pxNeoGFP-*LmjF21*.*0725*; HYG/PAC(+): Null mutant with addback pxNeoGFP-*LmjF*.*21*.*0725*; +/PAC/ (V) *L*. *major* carrying *Leishmania* expression vector pXNeoGFP. **(C)** Growth curve of *L*. *major* WT and mutant promastigotes (+/+: WT; +/PAC/ (+): heterozygous cell line with addback pxNeoGFP-*LmjF21*.*0725*; HYG/PAC(+): Null mutant with addback pxNeoGFP-*LmjF*.*21*.*0725*; +/PAC/ (V) *L*. *major* carrying *Leishmania* expression vector pXNeoGFP.

To confirm that *LmjF*.*21*.*0725* encodes for a protein expressed in the parasite, an immunofluorescence imaging experiment was performed based on the affinity-purified anti-LmjF.21.0725 antibody, indicating the cytoplasmic distribution of the LmjF.21.0725 antigen. Although present at low levels in the wild-type cells, the protein in the promastigote cytoplasm was observed in a similar distribution in the wild type (+/+) and in the LmjF.21.0725 transfectants evaluated (Fig 7A and 7B). Curiously, the low endogenous levels of this protein in *Leishmania* promastigotes has been similarly observed in wild-type *E*. *coli* cells [42]. The *in vitro* proliferation capacity of the transfectants, as indicated by the promastigote growth profile, showed no significant difference compared to the WT *L*. *major* (Fig 7C).

## Discussion

In the present report, an *in silico* analysis of the deduced sequence of LmjF.21.0725 indicated that the hypothetical protein does have a conserved signature domain belonging to the NAT superfamily. The reverse genetics analyses conducted indicate that the gene may be essential for promastigote survival.

One member of the NAT superfamily is the leucyl, phenylalanyl tRNA-protein transferase-like protein (LFTR-like), a close relative of the nonribosomal peptidyltransferase [23]. The enzyme L/F transferase plays a key role in bacterial post-translational modification as part of the N-end rule pathway and has been well characterized in *E*. *coli* [42]. The N-end rule is an evolutionarily conserved pathway that plays a central role in the survival of organisms submitted to a stressful microenvironment; it modifies the stability of regulatory proteins [43]. Although regulated proteolysis in *Leishmania* has been explored by others [44, 45], no aminoacyl-tRNA-protein transferase has been identified or characterized. Our analysis of the predicted structure of LmjF.21.0725 suggests that it is structurally highly similar to the L/F transferase of *E*. *coli*, despite limited overall amino acid sequence homology (sequence similarity of 30%, 17% identity). A shared limited sequence homology between L/F transferase of *E*. *coli* and LmjF21.0725, makes it difficult to predict the active site, however a large hydrophobic pocket was detected in our predicted model, that might be the active site (S5 Fig). We must bear in mind the limitations of computational sequence and structural analyses and the need for experimental evaluation to access the protein function.

Interestingly, a protein homologue from *P*. *falciparum* has been characterized with a structure similar to the bacterial L/F transferase but with the catalytic activity of an arginyl-tRNA protein transferase [22]. Our analysis showed limited overall sequence homology between the arginyl-tRNA protein transferase of *P*. *falciparum* and LmjF.21.0725 (36% similarity, 20% identity). Interestingly, the sequence conservation was high among the homologues of LmjF.21.0725 from various species of *Leishmania*, different from the higher divergence noted among putative L/F transferases from various *Plasmodium* species. We suggest that LmjF.21.0725 might be a phylogenetic marker capable of differentiating the protist parasite into respective classes and *Leishmania* species into the subgenus *Leishmania* and *Viannia*, as two distinct monophyletic lines. Nevertheless, the 3D model and primary sequence analyses show reasonable similarity/identity to the *E*. *coli* sequence and could be a limiting factor to differentiate species. This evidence is preliminary and further investigation must be conducted to confirm the hypothesis.

To conduct functional studies of the role of the *Lmj*.*F21*.*0725* product, we first attempted to disrupt both alleles. We obtained the heterozygous line but did not succeed in generating a double knockout. Hypothetically, this result indicated the possible essentiality of the gene for the promastigotes of *L*. *major*. This hypothesis was reinforced after obtaining the double replacement of two alleles with selectable markers in the strain transfected with the plasmid expressing LmjF.21.0725 [46, 47]. The generated transfectants and wild type promastigotes presented a similar profile of *in vitro* proliferation. Moreover, to confirm the presence and distribution of the hypothetical protein encoded by *LmjF*.*21*.*0725*, we exploited an anti-peptide antibody against the predicted amino acid chain of the coded protein. Immunofluorescence analysis confirmed the presence of a very low level of the expected protein in the wild-type *L*. *major* promastigotes. These results corroborate a previous report showing very low levels of the expression of endogenous LFTR in *E*. *coli* [42, 48]. Interestingly, even after testing various strategies, vectors and strains, we never succeeded in expressing LmjF.21.0725 in heterologous systems such as *E*. *coli* and *S*. *cerevisiae*; only the *L*. *tarentolae* expression system gave satisfactory results. Nevertheless, the amount of protein obtained was too low to establish the biochemical assay necessary to confirm its enzymatic activity. The purified protein was confirmed by Western blot analysis. The SDS-PAGE-purified rLmjF.21.0725 was further subjected to mass spectrophotometry, which confirmed its identity and validated it as a product of the studied protein encoding ORF (S4 Fig).

Analysis of the wild type and transfectant *LmjF*.*21*.*0725* locus anatomy by Southern blotting indicated that an unexpected recombination event occurred in the endogenous locus related to the PAC insertion event. Southern blot analysis of this transfectant genomic DNA digested with *Sal*I, a restriction enzyme with a predicted internal site in the PAC coding sequence, revealed an unexpected higher molecular weight band (7.5–8 Kb), which would be only explained by the lack of cleavage at the referred restriction site. The conditional null mutant was shown to have an additional copy of endogenous *LmjF*.*21*.*0725;* these results were consistently observed with 4 independent clones. A BLAST-n search using the *LmjF*.*21*.*0725* sequence against the *L*. *major* genome deposited at the TriTrypDB showed no additional copies of the gene. We further investigated possible ploidy or somy changes using pulsed field gel electrophoresis, but no differences were observed in the different lines, indicating no ploidy or full-chromosome copy number modifications. Our investigation does not rule out smaller regions of altered chromosomal structure, as it is known that events of partial somy or complete ploidy changes while inactivating an essential gene have been reported for *Leishmania* [47, 49–52]. Interestingly, the maintenance of two wild type alleles has been reported in *L*. *infantum* (GSH1); in this case, a single allele replacement resulted in the emergence of an extra copy of the gene [53]. Furthermore, it has been shown that aneuploidy in *Leishmania donovani* is associated with the adaptability of the parasite [54].

The essentiality of *LmjF*.*21*.*0725* in the promastigotes of *L*. *major* was also assessed via analysis of the dynamics of passive loss of the plasmid in the absence of the drug of selection, after serial passages using five independent conditional null mutants. The results corroborate previous results that suggested that the presence of *LmjF*.*21*.*0725* is essential for the survival of *L*. *major* promastigotes.

Essential pathways in parasites are key to the validation of new drug leads [55; 56], and genes playing essential roles in the viability of the *Leishmania* parasite are targeted for the discovery of new drugs [57–59]. Despite significant attempts, novel and safer drugs against leishmaniasis have yet to be developed. The available pentavalent drugs are effective, but their toxicity and the emergence of parasite resistance have led to an urgency in the identification and characterization of novel molecular targets. The *Leishmania major* genome has been sequenced, assembled and thoroughly annotated, representing a relevant resource for exploration of drug target candidates. Nevertheless, many genes do not yet have a predicted function and are assigned as hypothetical proteins encoding genes. Among them, several potential drug targets have been identified using *in silico* approaches [60–63]. Interestingly, a clpS N-end rule adaptor homologue of bacteria was recently characterized from *P*. *falciparum*, which has also shown expanded substrate specificity and is suggested as a novel target for anti-malarial drugs [64–65]. Regulated proteolysis involving the proteasome plays critical roles in the biology and virulence of parasites and is hence suggested as a valid drug target for sleeping sickness and malaria [66]. *Leishmania*-specific ubiquitin-related modifier (Ufm1) protein, which was shown to be functional and involved in the pathogenesis of *L*. *donovani*, is essential for cell division at the amastigote stage [67,68]. To the best of our knowledge, a *LmjF*.*21*.*0725* homologue has not yet been characterized from any species of *Leishmania*, and the available literature falls short of revealing any potential role in the aminoacyl-tRNA-protein transferase pathway in *Leishmania* cytodifferentiation and adaptation within the host macrophage. This study is the first *in silico* analysis to suggest the presence of a conserved putative aminoacyl-tRNA-protein transferase in *Leishmania*. Thus far, no attempts have been made to determine natural substrates or other biochemical properties of this enzyme. Experimental investigation is required to establish the catalytic activity of the LmjF.21.0725 protein in *Leishmania* and its putative role in virulence and pathogenesis.

In conclusion, the current report is a stepping stone for future research. The hypothetical protein LmjF.21.0725 is a structural homologue of N-acyl aminotransferase (NAT) superfamily proteins, it is highly conserved among the *Leishmania* species, and more importantly it is essential for parasite survival as a promastigote. Therefore, it is relevant to further investigate if this protein is also essential for amastigotes survival *in vivo*.

## Supporting information

S1 Fig Strategy for double replacement of endogenous *LmjF21*.0725 by selectable markers. (A) Schematic diagram showing strategy of LmjF21.0725 in *L*. *major*. (B) & (C) Vectors used for homologous recombination at Lmj21.0725 locus to replace endogenous gene with selectable markers: Hygromycin Phosphotransferase (HYG, 5.5 kb) and Puromycin Acetyl Transferase (PAC, 4.8 kb), respectively. (D) and (E) Targeting fragments obtained by restriction digestion of depicted vectors with Apa*I*/Stu*I* (2.4 kb; HYG) and Not*I* digestion (2 kb; PAC), respectively.
(TIF)

S2 Fig Overall quality assessment of the predicted 3D model of LmjF.21.0725 using Phyre2 investigator. (TIF)

S3 Fig *In silico* prediction of antigenic peptide. **(A)** Overall antigenicity of the LmjF.21.0725 indicating region selected for antigenic peptides (prediction 1 to 4, corresponding to peptides 1 to 4); **(B)** Hydrophobicity plot of the LmjF.21.0725 sequence showing the amino-acid sequence location of used peptide- (Peptide 4 = LFTR4- used for the study).
(TIF)

S4 Fig Heterologous expression of LmjF.21.0725 in *Leishmania tarentolae*. **(A)** *In silico* pLEXSY-NEO-Lmj21.0725 circular construct. **(B)** Agarose gel showing *Swa*I digested fragment of linear cassette for integration in SSU locus (6 kb). **(C)** Western blot analysis of whole-cell lysate of independent transfectant confirming expression by Western blotting using anti-HIS antibody **(D)** Western blot of fractions eluted with 500 mM imidazole using anti-Lmj21.0725 antibody; W- wash, FT- flow-through lysate, WCL-whole-cell lysate of *L*. *tarentolae* transfectant. **(E)** Polyacrylamide gel (12%) showing the purified Lmj21.0725 (42 kDa) **(F)** Confirmation of rLmj21.0725 using trypsin-digested mass spec analysis showing alignment between different identified peptides and deduced amino-acid sequences of LmjF.21.0725.
(TIF)

S5 Fig Hydrophobicity surface and large pocket for active site prediction. (**A**) Hydrophobic surface (Red color) of the predicted model of LmjF21.0725 predicted by PyMOL. (**B**) Largest predicted pocket of LmjF21.0725 indicates the possible active site determined by Phyre2 server using the Phyre2 investigator suite **(C**) LmjF21.0725 sequence showing the amino acid residues involved in formation of the pocket (Red color).
(TIF)

S1 Table List of primers used for KO and heterologous expression constructs. (TIF)

S2 Table Summary of P-Blast results; percent identity of LmjF21.0725 amino acid sequence with homologue sequences of various protist species. (TIF)
