# Supplementary figures and images for: Insights on a putative aminoacyl-tRNA-protein transferase of Leishmania major
Source: PLoS One. 2018 Sep 12;13(9):e0203369. doi: 10.1371/journal.pone.0203369 (PMC6135404; doi:10.1371/journal.pone.0203369)

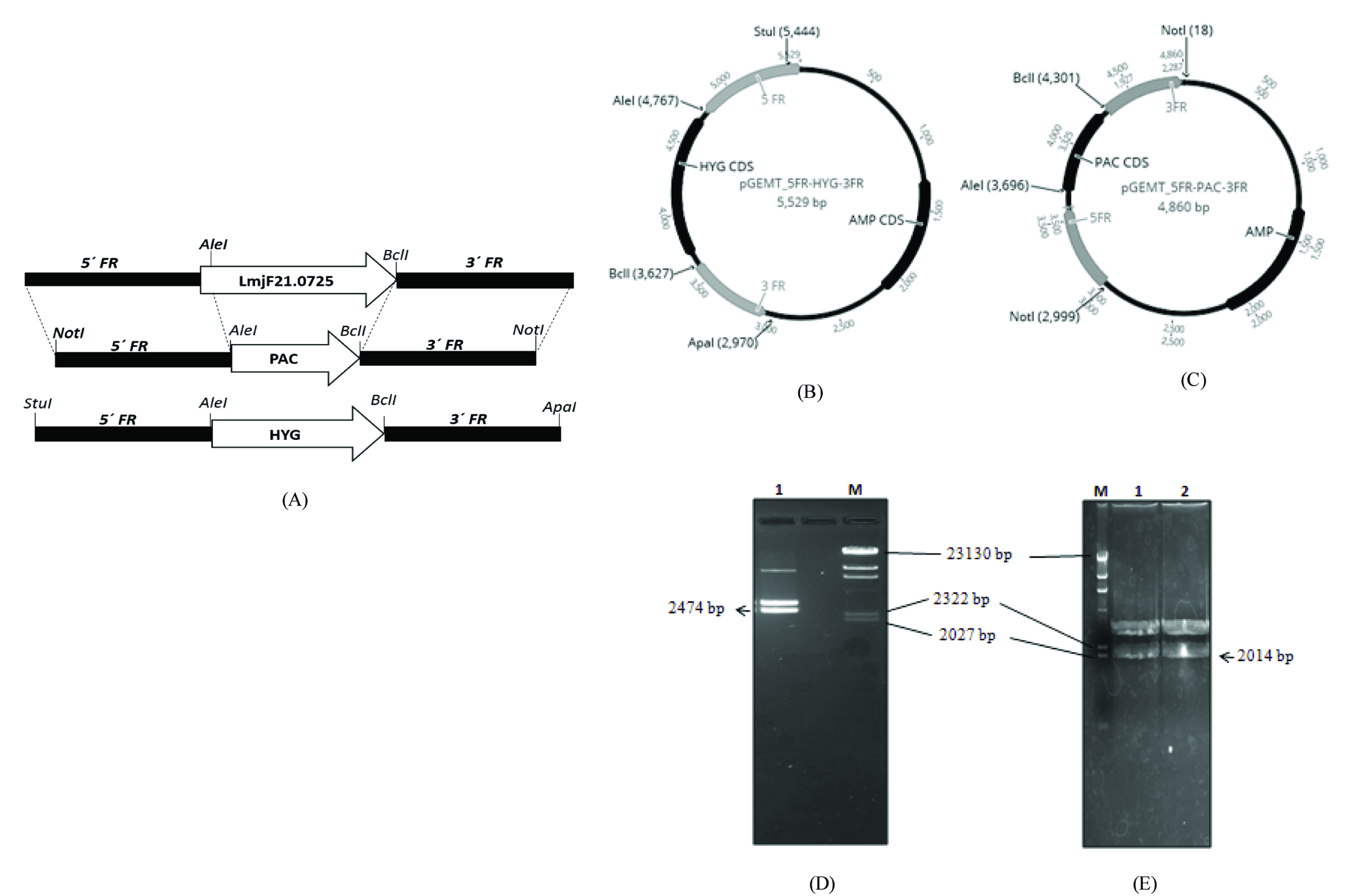

Supplement: S1 Fig — (A) Schematic diagram showing strategy of LmjF21.0725 in L. major. (B) & (C) Vectors used for homologous recombination at Lmj21.0725 locus to replace endogenous gene with selectable markers: Hygromycin Phosphotransferase (HYG, 5.5 kb) and Puromycin Acetyl Transferase (PAC, 4.8 kb), respectively. (D) and (E) Targeting fragments obtained by restriction digestion of depicted vectors with ApaI/StuI (2.4 kb; HYG) and NotI digestion (2 kb; PAC), respectively. (TIF) [file pone.0203369.s001.tif]

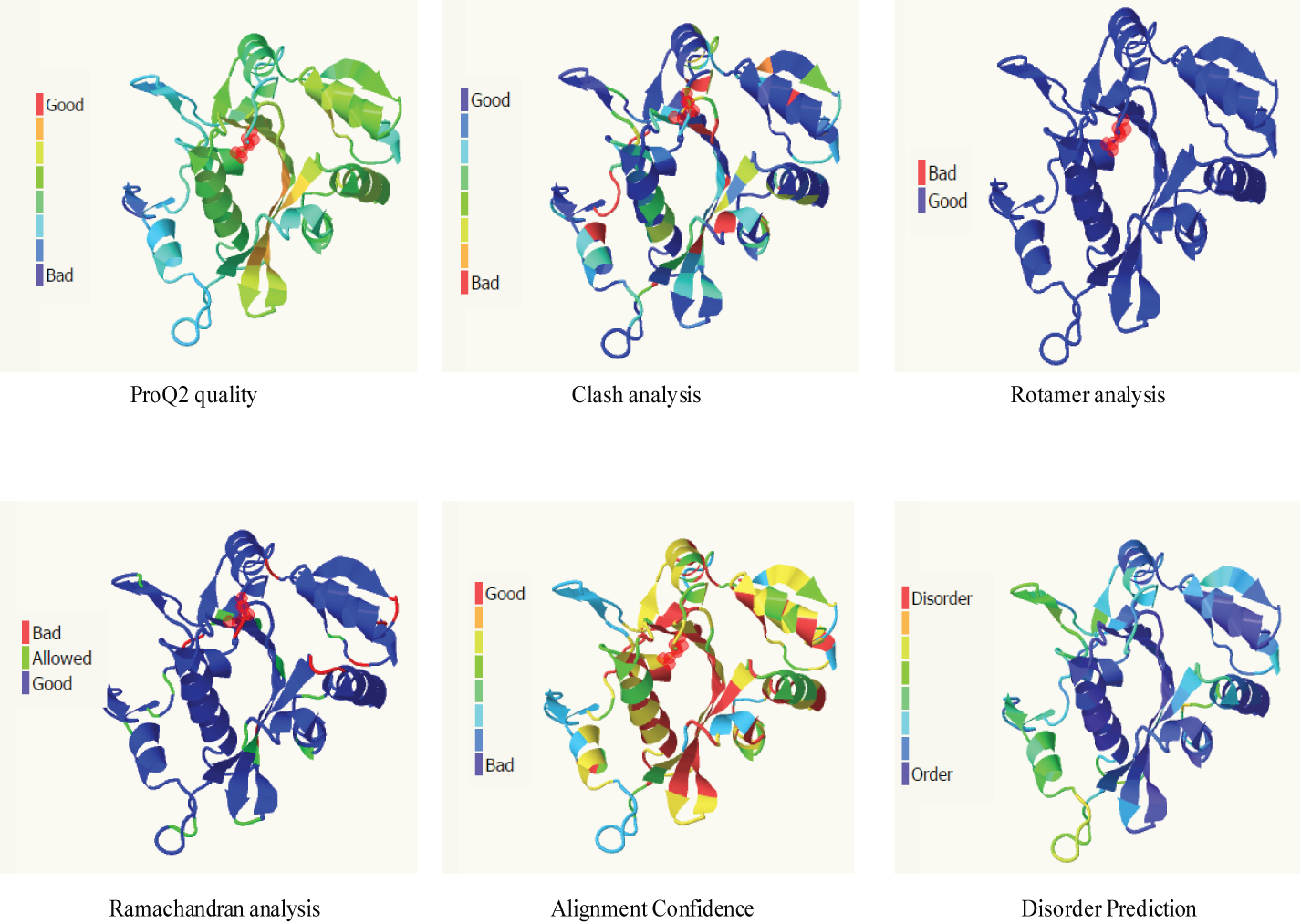

Supplement: S2 Fig — (TIF) [file pone.0203369.s002.tif]

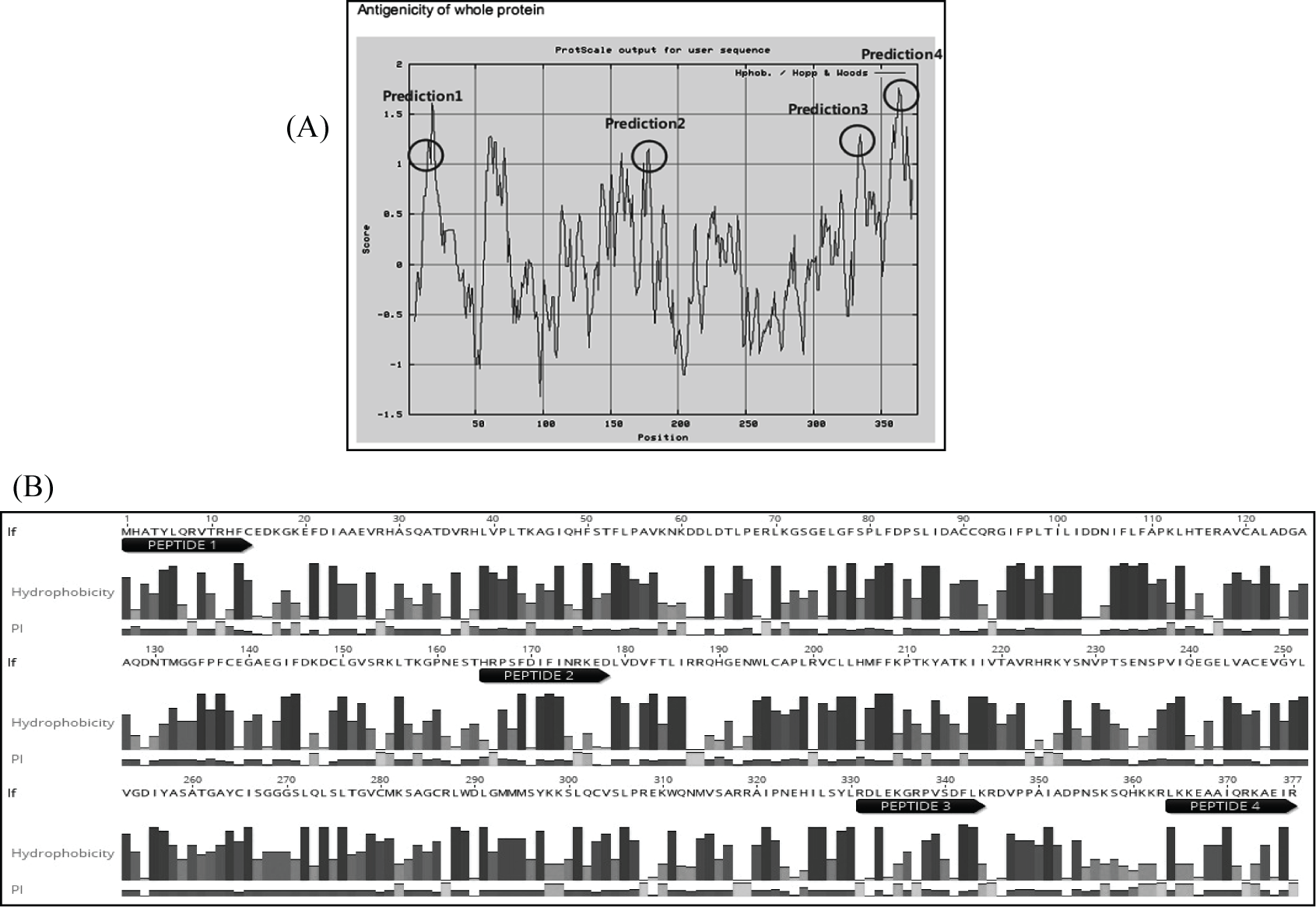

Supplement: S3 Fig — (A) Overall antigenicity of the LmjF.21.0725 indicating region selected for antigenic peptides (prediction 1 to 4, corresponding to peptides 1 to 4); (B) Hydrophobicity plot of the LmjF.21.0725 sequence showing the amino-acid sequence location of used peptide- (Peptide 4 = LFTR4- used for the study). (TIF) [file pone.0203369.s003.tif]

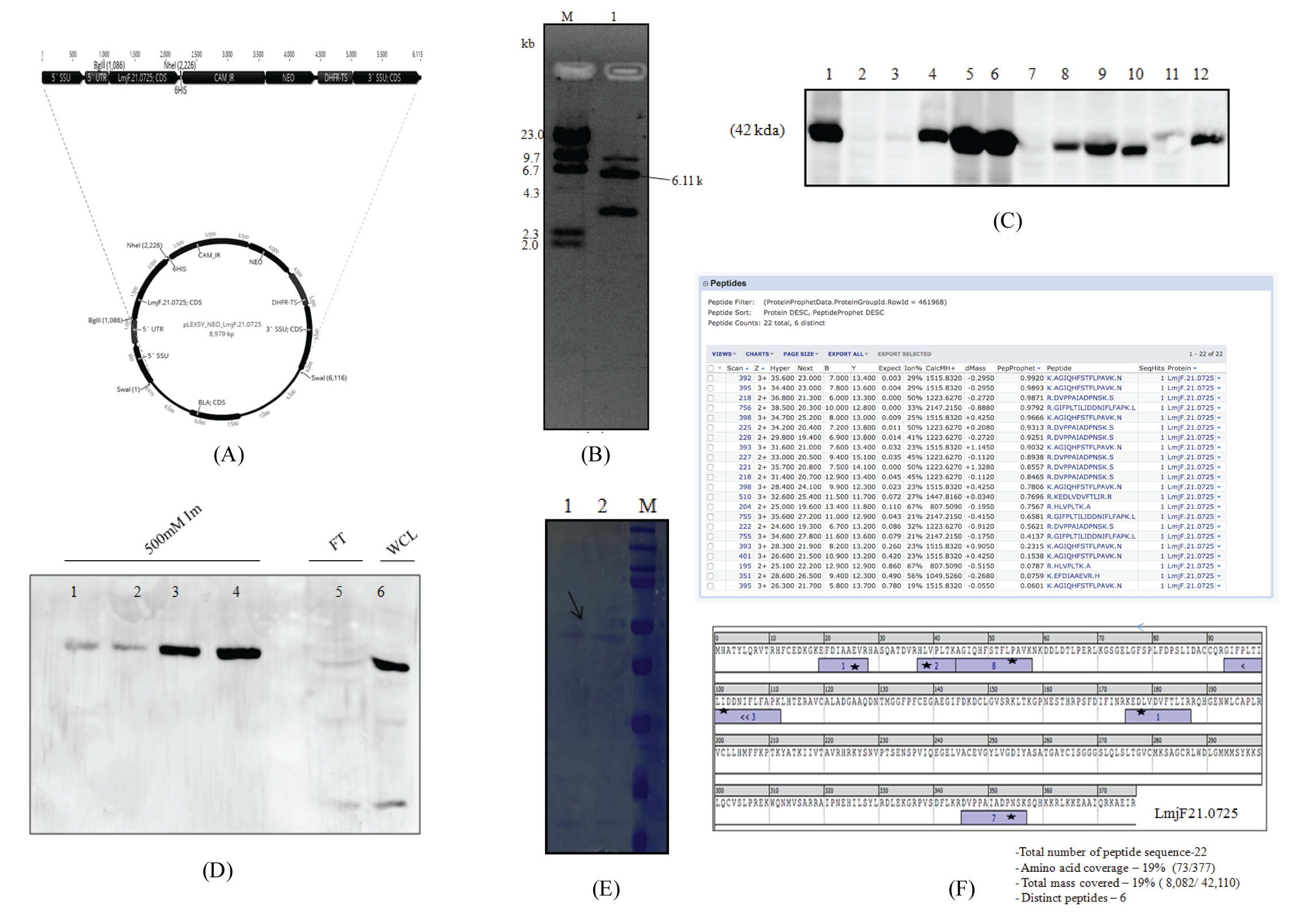

Supplement: S4 Fig — (A) In silico pLEXSY-NEO-Lmj21.0725 circular construct. (B) Agarose gel showing SwaI digested fragment of linear cassette for integration in SSU locus (6 kb). (C) Western blot analysis of whole-cell lysate of independent transfectant confirming expression by Western blotting using anti-HIS antibody (D) Western blot of fractions eluted with 500 mM imidazole using anti-Lmj21.0725 antibody; W- wash, FT- flow-through lysate, WCL-whole-cell lysate of L. tarentolae transfectant. (E) Polyacrylamide gel (12%) showing the purified Lmj21.0725 (42 kDa) (F) Confirmation of rLmj21.0725 using trypsin-digested mass spec analysis showing alignment between different identified peptides and deduced amino-acid sequences of LmjF.21.0725. (TIF) [file pone.0203369.s004.tif]

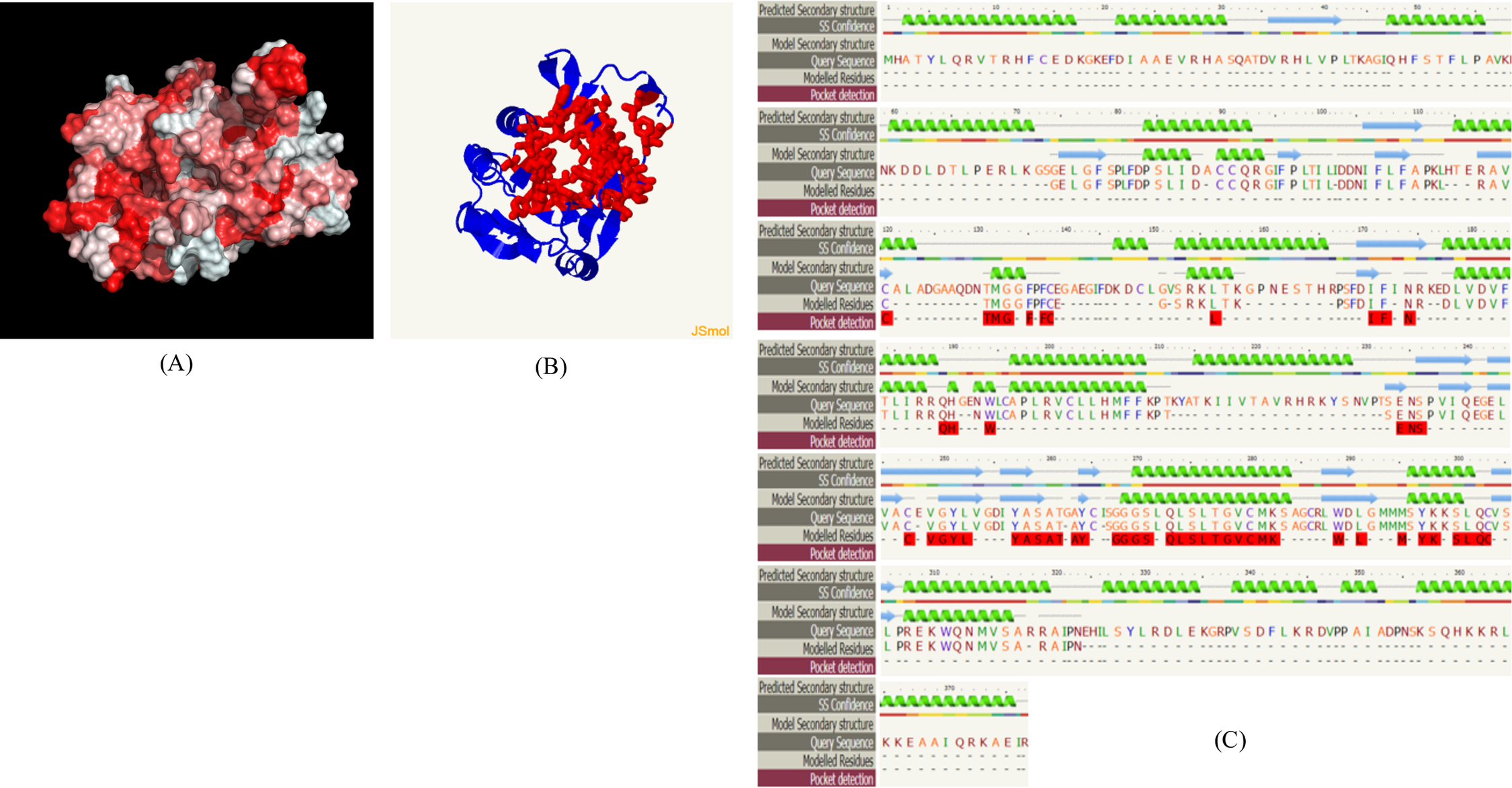

Supplement: S5 Fig — (A) Hydrophobic surface (Red color) of the predicted model of LmjF21.0725 predicted by PyMOL. (B) Largest predicted pocket of LmjF21.0725 indicates the possible active site determined by Phyre2 server using the Phyre2 investigator suite (C) LmjF21.0725 sequence showing the amino acid residues involved in formation of the pocket (Red color). (TIF) [file pone.0203369.s005.tif]

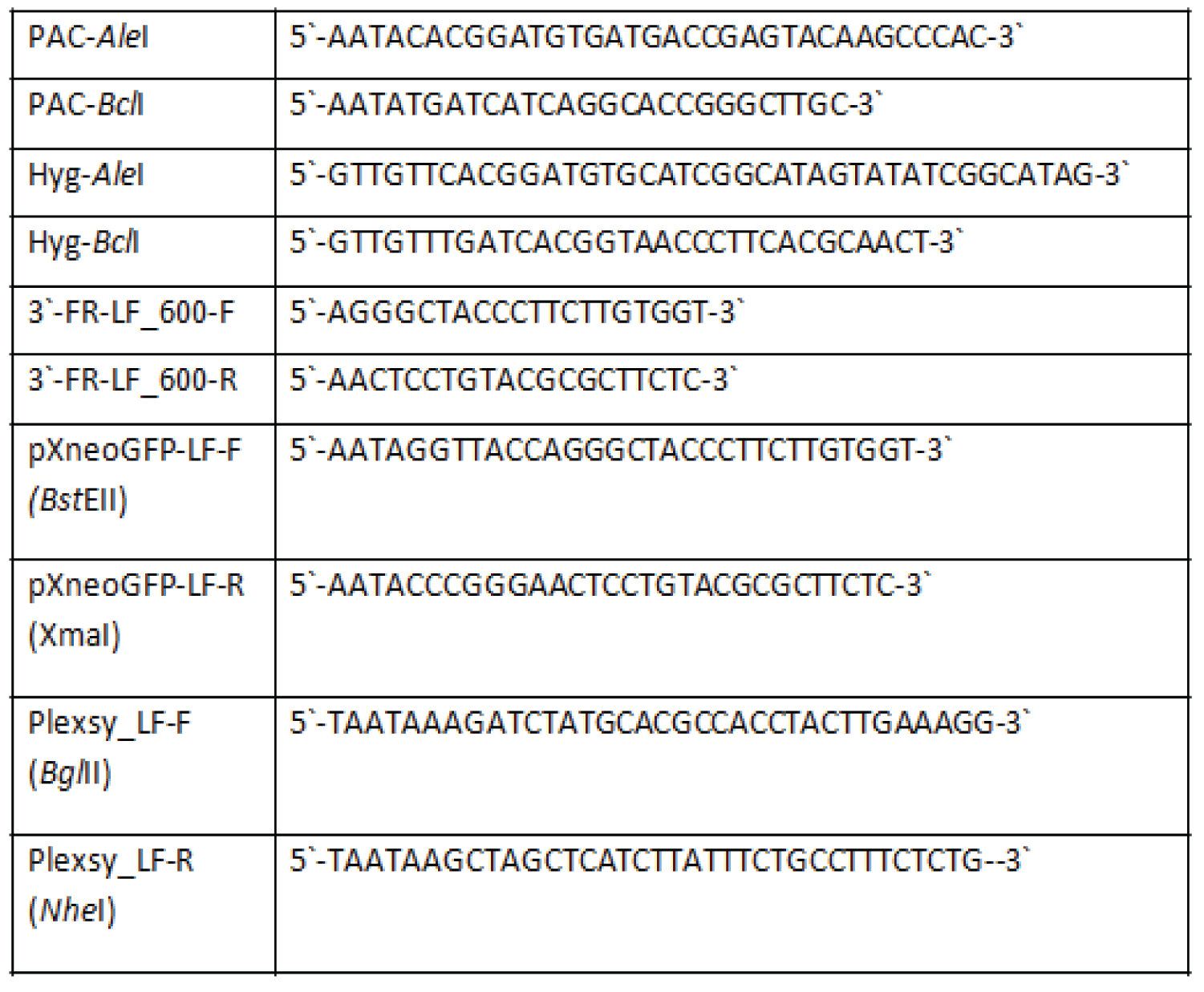

Supplement: S1 Table — (TIF) [file pone.0203369.s006.tif]

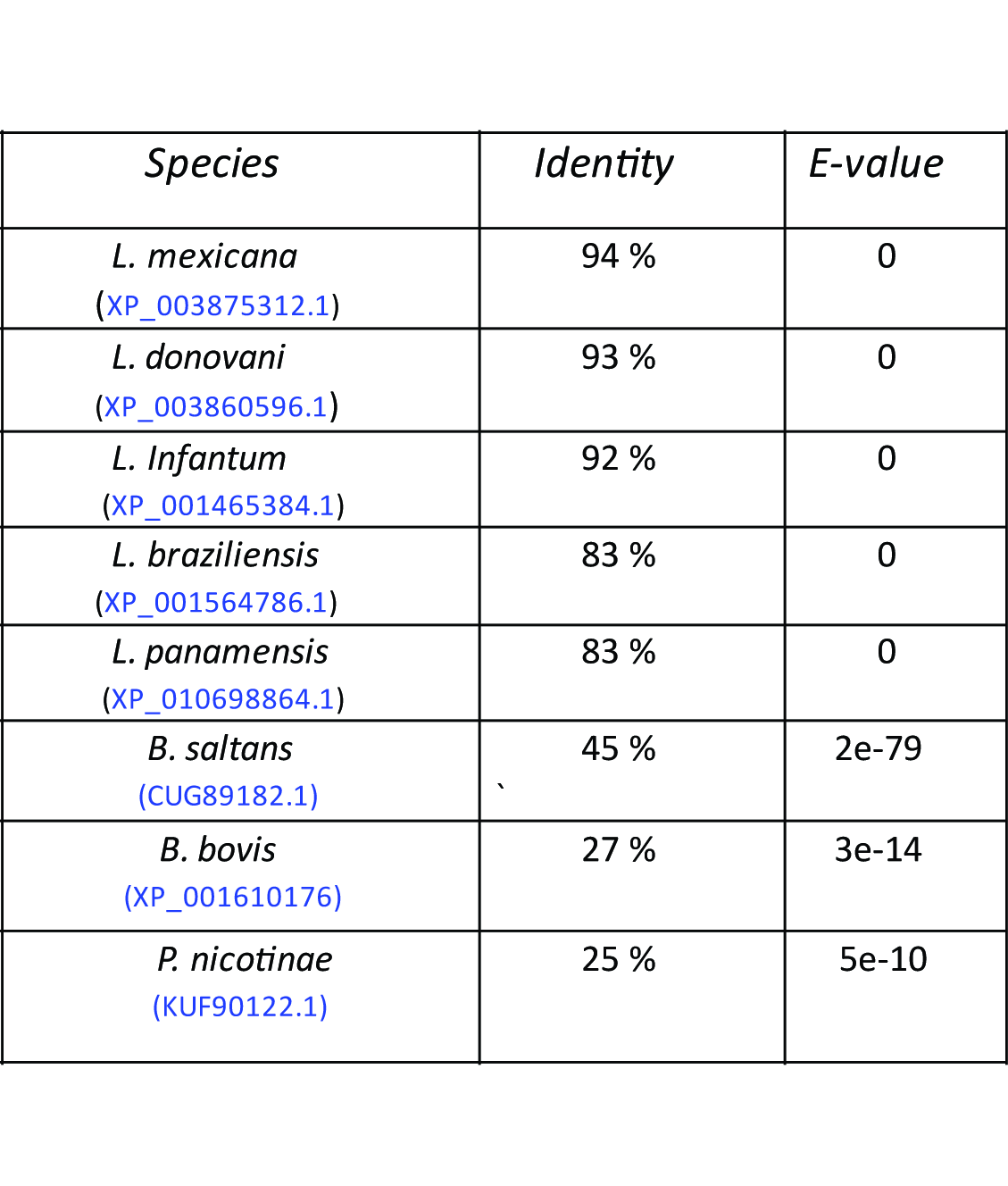

Supplement: S2 Table — (TIF) [file pone.0203369.s007.tif]
